# Supplementary material for: Design, Synthesis, and Biological Evaluation of 5,6,7,8-Tetrahydrobenzo[4,5]thieno[2,3-d]pyrimidines as Microtubule Targeting Agents
Source: Molecules. 2022 Jan 5;27(1):321. doi: 10.3390/molecules27010321 (PMC8747035; doi:10.3390/molecules27010321)
Supplement: Supplementary file 1 [file molecules-27-00321-s001.zip › molecules-1433106-supplementary.pdf]

Article

Design, Synthesis, and Biological Evaluation of 5,6,7,8-Tetrahydrobenzo[4,5]thieno[2,3-*d*]pyrimidines as Microtubule Targeting Agents

Farhana Islam [1], Arpit Doshi [1], Andrew J. Robles [2,3], Tasdique M. Quadery [1], Xin Zhang [1], Xilin Zhou [1], Ernest Hamel [4], Susan L. Mooberry [2,3,*] and Aleem Gangjee [1,*]

[1] Division of Medicinal Chemistry, Graduate School of Pharmaceutical Sciences, Duquesne University, 600 Forbes Avenue, Pittsburgh, PA 15282, USA; islamf@duq.edu (F.I.); doshia@duq.edu (A.D.); quaderyt@duq.edu (T.M.Q.); zhangx@duq.edu (X.Z.); zhoux@duq.edu (X.Z.)

[2] Department of Pharmacology, University of Texas Health Science Center at San Antonio, 7703 Floyd Curl Drive, San Antonio, TX 78229, USA; roblesa3@uthscsa.edu

[3] Mays Cancer Center, University of Texas Health Science Center at San Antonio, 7703 Floyd Curl Drive, San Antonio, TX 78229, USA

[4] Molecular Pharmacology Branch, Developmental Therapeutics Program, Frederick National Laboratory for Cancer Research, Division of Cancer Treatment and Diagnosis, National Cancer Institute, National Institutes of Health, Frederick, MD 21702, USA; hamele@dc37a.nci.nih.gov

* Correspondence: mooberry@uthscsa.edu (S.L.M.); gangjee@duq.edu (A.G.)

Supplementary Materials

Docking scores were measured using Schrödinger Maestro suite (Schrödinger, LLC, New York, NY, 2020-2).

Table S1. Docking scores.

	**Docking scores (kcal/mol)**
**1**	-10.15
**2**	-10.11
**3**	-10.10
**4**	-10.89
**5**	-10.72
**6**	-10.52
**7**	-10.68
**8**	-10.55
**9**	-9.68
**10**	-10.42
**11**	-9.75
**12**	-10.40
**13**	-10.45
**14**	-9.75
**Colchicine**	-11.02

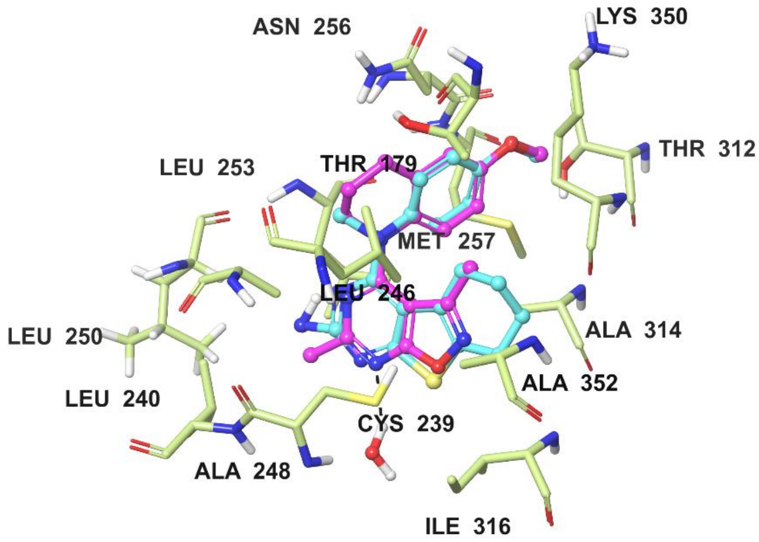

Figure S1. Superposition of 6BS2 pyrimidine ligand with compound **4.**
